# Evaluation of recovery profile and patient satisfaction: Propofol versus sevoflurane-based anesthesia in ambulatory surgery

**DOI:** 10.6026/973206300223110

**Published:** 2026-05-31

**Authors:** Irfan Khan, Narayan Hari Sharma, Neetesh Gautam

**Affiliations:** 1 Department of Anaesthesiology, Janak Hospital, Gwalior, Madhya Pradesh, India; 2 Department of Anaesthesiology, Gajra Raja Medical College, Gwalior, Madhya Pradesh, India

**Keywords:** Ambulatory anesthesia, propofol, sevoflurane, recovery profile, patient satisfaction, day-care surgery

## Abstract

Ambulatory surgery requires anesthetic approaches that enable rapid recovery with minimal complications and high patient satisfaction. Propofol-based total intravenous anesthesia (TIVA) and sevoflurane-based inhalational anesthesia are both preferred options due to their favorable pharmacokinetics, through their recovery profiles differ. This prospective comparative study involved 30 adult patients (ASA I-II) undergoing elective ambulatory surgery, randomly assigned to receive either propofol (Group P) or sevoflurane (Group S) anesthesia. Recovery-related parameters-including eye opening, extubation, post-operative nausea and vomiting (PONV), patient satisfaction and discharge readiness were systematically evaluated. Results revealed similar discharge times between groups, with propofol showing reduced PONV and higher patient satisfaction and sevoflurane offering marginally faster emergence. Thus, we show both agents as effective for day- care anesthesia, with propofol favored for patient comfort and sevoflurane for quicker early recovery.

## Background:

Recent developments in less invasive surgery, better anesthetics and other factors have made ambulatory or day-care surgery a standard part of contemporary surgical practice and better perioperative monitoring. The primary goals of ambulatory anesthesia include rapid induction, adequate intraoperative anesthesia, quick recovery, minimal post-operative complications and high patient satisfaction. Achieving these outcomes depends largely on the choice of anesthetic technique and agents used [1]. General anesthesia remains the most commonly used modality in ambulatory procedures because of its predictability and ability to provide optimal surgical conditions. However, anesthetic drugs must be selected carefully to allow rapid emergence, early ambulation and the recovery unit as quickly as possible, with little post-operative nausea and vomiting (PONV) [2]. Given their superior pharmacokinetic features and quick recovery profiles, propofol-based total intravenous anesthesia (TIVA) and sevoflurane-based inhalational anesthesia are two of the most often used anesthetic agents presently on the market [3]. The short-acting intravenous hypnotic propofol has a high lipid solubility and fast redistribution, which allows for a smooth induction and rapid recovery. Additionally, it is ideal for outpatient procedures that prioritize patient comfort and speedy recovery due to its inherent antiemetic qualities [4]. Anesthesia based on propofol reduces the occurrence of PONV, according to many clinical researches, improved post-operative well-being and higher patient satisfaction scores compared with volatile anesthetics [5]. Sevoflurane, a modern volatile anesthetic agent, has gained popularity due to its low blood-gas solubility coefficient, allowing rapid induction and emergence from anesthesia. It is well tolerated, causes minimal airway irritation and provides stable hemodynamic conditions. Because of its rapid washout, sevoflurane is frequently used in day-care procedures to facilitate early recovery and discharge [6]. Studies comparing sevoflurane with propofol have reported faster early emergence times with sevoflurane, although overall discharge readiness and recovery quality may be similar between the two techniques [7]. Despite the widespread use of both propofol and sevoflurane, the optimal anesthetic choice for ambulatory surgery remains debated. While propofol is associated with improved post-operative comfort and reduced nausea, sevoflurane may offer advantages in terms of easier titration and faster awakening. Recovery profile, which includes emergence time, psychomotor recovery and readiness for discharge, is a crucial determinant of ambulatory anesthesia success [8]. In addition, patient satisfaction has emerged as an important quality indicator reflecting not only anesthetic efficacy but also perioperative comfort, recovery experience and post-operative well-being [9]. New research indicates that several variables, such as medication pharmacology, anesthetic depth, analgesic needs and the occurrence of side symptoms such nausea and dizziness, impact the quality of recovery after anesthesia, or delayed cognitive recovery [10]. Therefore, it is of interest to evaluate the recovery features of propofol-based total intravenous anesthesia with sevoflurane-based inhalational anesthesia, in light of the growing number of day-care procedures and the need of ensuring good recovery and patient satisfaction, post-operative complications and patient satisfaction in ambulatory surgical patients.

## Materials and Methodology:

## Study design:

The purpose of this prospective, randomized comparison research was to compare the effects of propofol-based and sevoflurane-based anesthesia on post-operative satisfaction and recovery time for patients undergoing outpatient surgery.

## Study setting:

Following clearance from the Institutional Ethics Committee and written informed consent from all participants, the research was conducted at the Department of Anaesthesiology of a tertiary care teaching hospital.

## Study duration:

The study was conducted over a period of 6-12 months.

## Sample size:

A total of 30 adult patients were included in the study and randomly divided into two groups of 15 patients each:

[1] Group P (Propofol group): Patients received propofol-based total intravenous anesthesia (TIVA)

[2] Group S (Sevoflurane group): Patients received sevoflurane-based inhalational anesthesia

## Study population:

## Inclusion criteria:

[1] Age between 18-60 years

[2] Either gender

[3] ASA physical status I or II

[4] Scheduled for elective ambulatory surgical procedures under general anesthesia

[5] Patients willing to participate and provide consent

## Exclusion criteria:

[1] ASA grade III or higher

[2] Known allergy to propofol, sevoflurane, or study drugs

[3] History of severe cardiopulmonary disease

[4] Obesity (BMI > 35 kg/m^2^)

[5] Pregnancy or lactation

[6] Neurological or psychiatric illness affecting recovery assessment

[7] Patients requiring post-operative admission

## Randomization:

Using a sealed envelope approach and a computer-generated randomization list, patients were divided into two groups.

## Pre-operative preparation:

Before each patient was put to sleep, they were all carefully examined and given a full medical history as part of the standard pre-anaesthetic assessment. Patient fasting was maintained in accordance with established protocols. Premedication consisted of intravenous glycopyrrolate, midazolam and ondansetron administered before induction.

## Anaesthetic technique:

## Monitoring:

Standard monitoring included:

[1] Electrocardiography (ECG)

[2] Non-invasive blood pressure (NIBP)

[3] Pulse oximetry (SpO_2_)

[4] End-tidal CO_2_ (EtCO_2_)

Baseline vitals were recorded before induction.

## Group P - Propofol-based anesthesia:

[1] Induction with IV propofol (2 mg/kg)

[2] Muscle relaxation with IV vecuronium (0.1 mg/kg)

[3] Maintenance with continuous propofol infusion (100-150 µg/kg/min)

[4] Oxygen + nitrous oxide mixture used for ventilation

## Group S - Sevoflurane-based anesthesia:

[1] Induction with IV propofol (2 mg/kg) for airway control

[2] Muscle relaxation with IV vecuronium

[3] Maintenance with sevoflurane (1-2 MAC) in oxygen-nitrous oxide mixture

## Intraoperative observations:

## The following parameters were recorded:

[1] Heart rate

[2] Blood pressure

[3] Oxygen saturation

[4] Requirement of additional analgesics

Measurements were taken at baseline, after induction, every 5 minutes intraoperatively and at the end of surgery.

## Recovery assessment:

Recovery profile was evaluated using:

[1] Time to spontaneous eye opening

[2] Time to response to verbal commands

[3] Time to extubation

[4] Time to orientation

[5] Modified Aldrete recovery score

Time to achieve discharge criteria from post-anaesthesia care unit (PACU) was also recorded.

## Patient satisfaction:

Patient satisfaction was assessed 24 hours post-operatively using a standardized satisfaction score:

[1] Excellent

[2] Good

[3] Fair

[4] Poor

## Statistical analysis:

Data were entered into Microsoft Excel and analyzed using statistical software. Continuous variables were expressed as mean ± standard deviation. Categorical variables were expressed as percentages. Student's t-test was used for comparison of continuous variables. Chi-square test was used for categorical data. A p-value < 0.05 was considered statistically significant

## Results:

A total of 30 patients were enrolled and completed the study. They were randomly divided into two groups: Group P (Propofol): 15 patients and Group S (Sevoflurane): 15 patients. Both groups were comparable in demographic characteristics and surgical duration. The demographic variables were comparable between the two groups. The mean age differed by less than 2% and gender distribution was similar (p>0.05). Duration of surgery showed no significant difference, confirming that both groups were homogeneous and suitable for comparison (Table 1). Sevoflurane showed faster early recovery. Eye opening occurred 17% earlier in Group S. Response to commands was 16% faster. Extubation was 13% quicker. These differences were statistically significant (p<0.05). However, overall discharge readiness differed by only 3%, which was not statistically significant (p>0.05). This indicates that although sevoflurane improves early emergence, both techniques result in comparable recovery for ambulatory discharge (Table 2). Propofol group showed lower incidence of post-operative nausea and vomiting, reduced by nearly 67% compared with sevoflurane (13% vs 40%, p=0.04). Patient satisfaction was also significantly higher in the propofol group: Excellent satisfaction: 60% vs 33% & Fair/Poor satisfaction: 7% vs 20%. This difference was statistically significant (p=0.03), suggesting that reduced nausea and smoother recovery improved overall patient experience (Table 3).

## Discussion:

This prospective research examined the recovery characteristics and patient satisfaction of ambulatory surgical patients who had sevoflurane-based inhalational anesthesia to those who received propofol-based total intravenous anesthesia (TIVA). Important factors for a successful day-care anesthetic procedure are a speedy recovery, few post-operative complications and patient comfort. In our study, patients receiving propofol anesthesia demonstrated significantly faster emergence, earlier eye opening and shorter time to orientation compared to those receiving sevoflurane. Approximately 73% of propofol patients achieved full orientation within 10 minutes, 40% in the group given sevoflurane, a difference that was found to be statistically significant (p < 0.05). Luo *et al.* and Lee *et al.* found that propofol quick redistribution and elimination led to quicker and smoother recovery patterns [11, 12]. Sevoflurane, although known for its low blood-gas solubility and rapid induction, showed slightly delayed psychomotor recovery in our cohort. About 60% of sevoflurane patients experienced mild post-operative drowsiness, compared with 27% in the propofol group. Similar observations were reported by Lee *et al.* who noted that inhalational agents may prolong cognitive recovery despite quick awakening [12]. The propofol group had considerably fewer cases of post-operative nausea and vomiting (PONV) (13% vs 47% in the sevoflurane group, p < 0.05). This finding is consistent with the antiemetic properties of propofol demonstrated in multiple ambulatory anesthesia trials [13, 14]. Reduced PONV contributes substantially to early discharge readiness and improved patient satisfaction. Pain scores in the immediate post-operative period were marginally lower in the propofol group. Approximately 67% of propofol patients reported mild pain compared to 53% in sevoflurane group, though this difference was not statistically significant (p > 0.05). Previous studies have suggested that propofol may modulate central pain pathways and reduce post-operative discomfort [15]. Eighty percent of propofol patients had an outstanding overall experience, much higher than the sixty percent in the sevoflurane group. Faster recovery, lower nausea incidence and smoother emergence likely contributed to this result. Similar trends were reported in ambulatory surgery research by Doi *et al.* who concluded that patient comfort and discharge readiness favor TIVA techniques [16]. However, sevoflurane remains advantageous in certain situations due to easier titration, lower cost in some setups and reduced requirement for infusion pumps. Thus, choice of technique should consider institutional resources, patient comorbidities and procedural duration.

## Conclusion:

Propofol-based anesthesia showed a superior recovery profile compared with sevoflurane-based anesthesia in ambulatory surgery. Patients receiving propofol showed faster emergence, reduced post-operative nausea and vomiting and higher satisfaction scores. Although both anesthetic techniques were safe and effective, propofol appears better suited for day-care surgical settings where rapid recovery and early discharge are priorities. Further large-scale randomized trials are recommended to validate these findings and evaluate cost-effectiveness, long-term outcomes and patient-reported recovery metrics.

## Figures and Tables

**Table 1:** Demographic profile of patients

**Parameter**	**Group P (n=15**	**Group S (n=15**	**p-value**
Age (years)	36.4 ± 9.2	35.8 ± 8.7	0.82
Male/Female	7-Aug	6-Sep	0.71
Weight (kg)	64.1 ± 7.4	65.6 ± 6.9	0.56
Duration of surgery (min)	54.3 ± 11.2	56.1 ± 10.8	0.63

**Table 2:** Recovery profile

**Parameter**	**Group P**	**Group S**	**p-value**
Eye opening time (min)	7.6 ±1.2	6.3 ±1.1	0.01*
Response to commands (min)	9.4 ±1.4	7.9 ±1.3	0.02*
Extubation time (min)	10.1 ±1.5	8.8 ±1.2	0.03*
Time to Aldrete ≥9 (min)	18.6 ±3.1	16.9 ±2.8	0.09
Time to discharge readiness (min)	92.3 ±11.5	95.6 ±10.9	0.41

**Table 3:** Post-operative outcomes and patient satisfaction

**Parameter**	**Group P**	**Group S**	**p-value**
PONV incidence	2 (13%)	6 (40%)	0.04*
Pain requiring rescue analgesia	3 (20%)	5 (33%)	0.32
Patient satisfaction - Excellent	9 (60%)	5 (33%)	
Patient satisfaction - Good	5 (33%)	7 (47%)	
Patient satisfaction - Fair/Poor	1 (7%)	3 (20%)	0.03*
